# Handgrip Strength Exercises Modulate Shoulder Pain, Function, and Strength of Rotator Cuff Muscles of Patients with Primary Subacromial Impingement Syndrome

**DOI:** 10.1155/2022/9151831

**Published:** 2022-08-30

**Authors:** Amal AlAnazi, Ahmad H. Alghadir, Sami A. Gabr

**Affiliations:** Department of Rehabilitation Sciences, College of Applied Medical Sciences, King Saud University, Riyadh, Saudi Arabia

## Abstract

**Background:**

Impingement syndrome was shown to be associated with shoulder pain in 44–70% of patients worldwide. It usually occurs due to imbalance and insufficient activation of the rotator cuff (RC) muscles.

**Aim:**

This study explores the relative effects of handgrip-strengthening exercises on shoulder function, pain, strength, and active range of motion as part of the treatment program for the patients with primary subacromial impingement syndrome.

**Materials and Methods:**

A total of 58 patients aged 18-50 years with primary subacromial impingement syndrome were randomly enrolled to participate in this single-blind randomized clinical trial. Out of them, only forty patients have eligibly matched the inclusion criteria and randomly assigned to one of two groups to undergo a standardized therapeutic program consisting of two sessions a week for 8 weeks. The control group prescribed ultrasound therapy, ice, and stretching exercises, while the experimental group followed the same program with the addition of handgrip-strengthening exercises (HGSE). Both patients of conventional therapy (control) and handgrip-strengthening exercises (experimental group) were advised to adhere also to stretching and HGSE exercises once a day at home for eight weeks. The outcomes were the shoulder function, pain intensity, muscle strength, and active range of motion of the shoulder joint.

**Results:**

Patients treated with conventional interventions plus handgrip-strengthening exercises showed the significant improvement over time in shoulder pain and function, strength of rotator cuff muscles, and pain-free range of motion forward flexion, abduction, and external and internal rotation through eight weeks in the experimental group compared to control patient group treated with conventional interventions. In addition, patients of both control and experimental groups showed no significant difference in the adherence to respective home-based stretching and HGSE exercises once a day at home for eight weeks.

**Conclusions:**

Adding handgrip-strengthening exercises to conventional intervention increases the efficacy of treatment for patients with primary subacromial impingement syndrome in terms of shoulder function, pain, muscle strength, and active range of motion.

## 1. Introduction

Shoulder dysfunction is a common complaint of patients visiting physiotherapy clinics, with 20–30% of the general population having been diagnosed with shoulder pain [1]. It was reported that shoulder pain comprises the most third prevalent musculoskeletal pain, following spinal and knee pain [2]. Previous research studies showed that impingement syndrome was estimated in 44–70% of patients with shoulder pain [2–4], which usually occurs due to imbalance and insufficient activation of the rotator cuff (RC) muscles [5].

The condition of subacromial impingement syndrome (SAIS) is a common shoulder pathology in general practice, which ranges from bursitis and degeneration of the RC tendons to full-thickness tendon tears [6–8]. SAIS was previously diagnosed as mechanical abrasion and compression of the RC muscles as they pass under the coracoacromial arch during arm elevation as inadequate subacromial space for clearance of the RC tendon leads to impingement [6–9]. Usually, impingement symptoms are classified as internal or external impingement based on the site of disorder, or primary or secondary impingement based on the cause of the problem [9–11].

Furthermore, SAIS was shown to be associated with some mechanical factors such as trauma, tension overuse, or improper force-coupling of the RC [12], and shoulder kinematic abnormalities can contribute to SAIS [12–14]. Functional factors that lead to the development of SAIS include an inefficient RC, capsular mobility impairment, and abnormal scapular motions [15]. Primary SAIS is one of subacromial syndromes which referred to an alteration in the patterns of muscle activation patterns, whereas inadequate external rotation of the humerus during any motion brings the greater tuberosity closer to the coracoacromial arch, thereby aggravating symptoms during arm elevation [16–18]. In addition to that, an extreme superior and anterior translation has been demonstrated to lead to the development of shoulder impingement syndrome and RC degeneration [12–14]. Although a reduction in RC and scapulothoracic muscles was reported during arm elevation in conditions of experimental acute pain applied to investigate the effects of acute pain on muscle activation using muscle functional magnetic resonance imaging (mfMRI), it remains unclear whether pain arises from the alteration of muscle activity or is secondary to this effect [2, 19].

Exercise therapy is the most effective treatment for subacromial impingement syndrome (SAIS) [20, 21]. Most exercise interventions applied for treating SAIS are significantly aimed at strengthening the RC muscles that stabilize the joint during movement and also act as prime movers [22]. Currently, insufficient evidence exists to validate specific exercise strategies for treatment of SAIS [23]. Essentially, shoulder movements are used to position and move the hand during fine tasks. Due to the muscle imbalance that exists in patients with SAIS, handgrip strength is concomitantly affected, and the positive correlation between handgrip and shoulder muscle strength has been demonstrated in previous studies [24–28]. Although strengthening the handgrip is an essential aspect of SAIS treatment [23–28], no previous studies have investigated the effects of including handgrip-strengthening exercises in treatment programs for patients with SAIS.

It was hypothesized that the addition of handgrip-strengthening exercises to a standardized conventional treatment for SAIS will produce superior results in terms of function, pain reduction, muscle strength, and pain-free range of motion (AROM) of the shoulder compared with standardized conventional treatment alone. Thus, the aim of this study was to investigate the relative effect of handgrip-strengthening exercises in terms of improvement of shoulder function, pain, strength, and pain-free active range of motion (AROM) in patients with primary SAIS. In this study, patients were classified into two groups: patients treated with standardized conventional treatment (conventional group) and patients treated with conventional therapy along with handgrip-strengthening exercises (HGSE). All patients were asked to adhere also to exercises at home once a day for eight weeks.

## 2. Materials and Methods

### 2.1. Subjects

**Figure 1:**
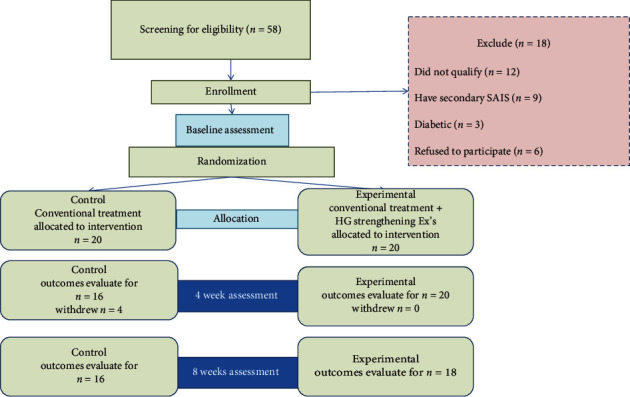
Flowchart of participants through each stage of the study.

A total of 58 patients aged 18-50 years from the King Abdul-Aziz Hospital & Oncology Center and the East Jeddah General Hospital, Jeddah, KSA, were recruited to participate in this single-blind randomized clinical trial. Patients diagnosed by a referred medical orthopedist as unilateral SAIS for less than one year with a reported pain intensity of 3 to 8 were included in this study. However, patients with a history of shoulder fracture or dislocation, osteophytes, or labral tear that precludes the ability to perform exercises of the upper extremities; a history of cardiac, neurological, or musculoskeletal disease; a history of shoulder, cervical, or thoracic surgery or patients who had hand or forearm dysfunction, rheumatoid disease, diabetes, malignancy, or pregnancy were excluded from this study. Only 38 patients who matched with the proposed inclusions completed conventional treatments and handgrip-strengthening exercise interventions for 8 weeks as reported in flow chart (figure 1).

Regarding the ethical guidelines of the 1975 Declaration of Helsinki, the present study was approved by the ethics committee of the College of Applied Medical Sciences, King Saud University (under the approval number: CAMS 039-3839, approval date: 10/12/2017). The study was also approved by the Research Ethics Committee of the Ministry of Health Directorate of Health Affairs, Jeddah (under the approval number: A00532, approval date: 15/01/2018).

In addition, the study was registered at ClinicalTrials.gov with the identifier number: NCT03468088. All participants were assigned a written informed consent before data collection. Research design, demographic, and clinical data of the participants are present in a flow chart (Figure 1 and Table 1, respectively).

### 2.2. Assessments of Physical Therapy Treatments

In this study, for the clinical assessment of impingement, standard physical therapy evaluations were performed as previously reported [29–31]. In these evaluations, impingement was clinically assessed in relation to the position of shoulder pain, the involvement of RC pathology as the primary cause of SAIS [29–31].

Patients were assigned randomly to clinical interventions to either conventional (control group) or handgrip-strengthening (experimental group) intervention [30, 31]. For the restoration of function in SAIS, patients attended supervised physiotherapy sessions twice a week for 8 weeks as previously reported [31]. In the present study, patients were advised to do only the exercises given as part of the study intervention and to avert adding any new upper body exercises. They were also instructed not to undergo other therapy during the study period.

#### 2.2.1. Conventional Intervention

In this intervention, firstly, patients with primary SAIS received ultrasound therapy (US). Each patient seated in an adjustable chair with back support and their feet on the floor; then US therapy was performed.

The chair was positioned beside a table, and the patient's shoulder rested on the table beside the body with the elbow flexed at 90°. A round-headed probe was placed in direct contact with the patient's skin over the shoulder joint. Ultrasound gel was applied to all surfaces of the head to reduce friction and assist in transmission of the ultrasonic waves. Pulsed therapeutic US was applied at a frequency of 3 MHz and intensity of approximately 1.5 W/cm^2^. Usually, no sensation of heat would be felt, but the intensity was reduced if the patient felt discomfort. A typical US treatment would take 8 minutes.

During treatment, the head of the US probe was kept in constant motion, which should ensure no discomfort to the patient. The application of ice is widely used for reducing pain in shoulder impingement syndrome [29–31]. The follow-up assessments were carried out at 4 and 8 weeks after the initiation of treatment. Stretching exercises including posterior shoulder muscle, pectoralis, seated thoracic spine extension, and sleeper stretches were performed under supervision also given as home program once a day for eight weeks as shown in Table 2. Every week, patients were asked to state the percentage of adherence to the home program-advised exercise and to indicate if they face any adverse effects.

#### 2.2.2. Handgrip-Strengthening Intervention

**Figure 2:**
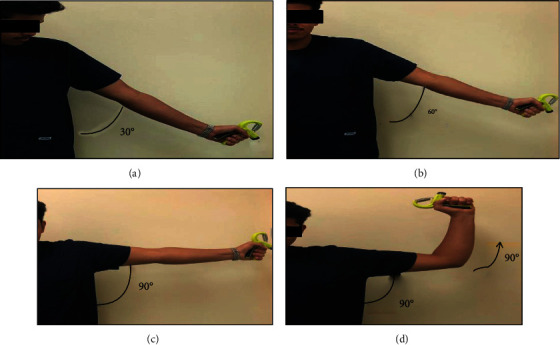
Handgrip-strengthening exercises using an adjustable heavy-grip handgripper at different sets (30°, 60°, and 90°); (a) 1^st^ and 2^nd^ weeks at 30°, (b) 3^rd^ and 4^th^ weeks at 60°, (c) 5^th^ and 6^th^ weeks at 90°, and (d) 7^th^ and 8^th^ weeks at 90°.

Handgrip-strengthening exercises (HGSE) were performed under therapeutic supervision in addition with the standardized conventional intervention for the patients with primary SAIS in experimental group as previously mentioned in the literature [24, 25, 32, 33]. An adjustable heavy-grip handgripper (El-Falah Sports House Co., China) was used to perform 10 repetitions at repetition maximum as shown in Figure 2. Patients were asked to perform the exercises in a standing position with their back against a wall, arm at either 30, 60, or 90° of abduction, and with 90° external rotations as in Figures 2(a)–2(d). In this position, patients performed three sets of 10 squeezes at 1 minute once a day. The resistance was reset to the 10 repetition maximum every 2 weeks by using the Epley formula: *w* (1 + *r*/30), assuming *r* > 1, where *r* is repetition every patient had his/her own adjustable heavy-grip handgripper.

The arm position was adjusted every 2 weeks as patient tolerance increased. In the first and second weeks, exercise were carried out at 30° of abduction, changing to 60° of abduction in the third and fourth weeks, and 90° of abduction in the fifth and sixth weeks (Figures 2(c) and 2(d)). Finally, exercises were performed at 90° of abduction with 90° external rotations in last two weeks. Also, patients were asked to perform the same HGSE exercises at home once a day for eight weeks.

### 2.3. Assessment of Outcome Measures

All patients in the conventional (control) and handgrip-strengthening-treated groups (experimental) were subjected for the estimation of pain intensity (VAS score), shoulder function (DASH score), muscle strength (internal/external), and pain-free active range of motion (ROM) measures at respective time intervals: baseline, 4 weeks, and 8 weeks of treatments.

#### 2.3.1. Shoulder Function and Strength Testing

Prior to strength testing, patients were asked to fill out DASH questionnaires, to apply marks to the point that they felt represented their perception of their current state. Isometric strengths of internal and external rotation were assessed using hand-held dynamometer (HDD) with the patient seated in the neutral position, maintaining the shoulder at 0° of abduction, holding the forearm in the neutral position with the elbow flexed at 90° [34].

**Figure 3:**
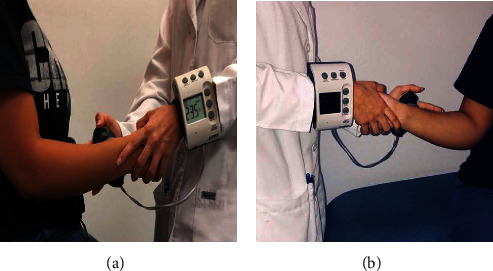
Strength of rotator cuff muscle measured by a hand-held dynamometer (HHD). (a) Internal rotation; (b) external rotation.

The curved-end attachment of the HHD was placed about 0.5 inches proximal to the ulnar styloid process. The participant was asked to perform a glenohumeral internal or external rotation motion, back-off to midrange and hold the position as shown in Figures 3(a) and 3(b). Participants held the positions while the therapist applied force through the dynamometer using the “make test” procedure, building to maximal tension in 2 seconds and holding the tension for 5 seconds. The dynamometer was held in place by matching the force exerted by the patient. If the individual was unable to sustain muscle contraction against resistance, the data was not recorded and the test was repeated as mentioned previously in the literature [34, 35]. Two measurements were taken for each motion with a 30-second rest between to allow muscle recovery. The means of the two trials of each strength test were used for data analysis.

#### 2.3.2. Range of Motion Assessments and Pain Intensity

**Figure 4:**
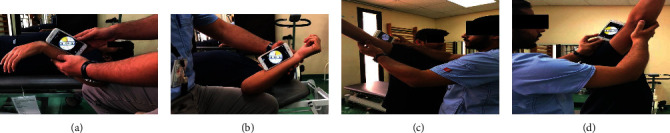
Measurement of ROM of shoulder joint using a smartphone inclinometer. (a) Internal rotation, (b) external rotation, (c) abduction, and (d) forward flexion.

Subsequently, assessment of shoulder joint AROM was carried out using the smartphone clinometer as shown in Figure 4. In the assessment of joint AROM at 90°, internal and external rotations of the shoulder with flexion and abduction were performed for each patient separately as shown in Figures 4(a)–4(d). This ensured correct technique and understanding before the AROM assessment was performed. Patients were instructed to perform the movement as far as they were able to without pain or trunk motion [36]. From the standing position, forward flexion was assessed by asking the patient to raise the arm straight up in front of them with the thumb pointing upwards as high as they were able (Figure 4(d)). Abduction was assessed by asking the patient to raise the arm to the side as high as they were able without pain or trunk motion (Figure 4(c)). The smartphone clinometer was placed proximal to the lateral epicondyle of the elbow joint [37]. From the supine position, the shoulder would be at 90° of abduction and the elbow at 90° of flexion, with neutral supination/pronation of the forearm (Figures 4(c) and 4(d)). The patient was asked to keep the elbow at 90° and move the forearm backward as far as they were able and then forwards as far as they were able. Assessment of internal and external rotation at 90° of abduction was achieved by placing the smartphone clinometer proximal to the ulnar styloid process [37]. Two measurements were taken for each movement, and the mean values were used for data analysis. Finally, assessment of pain intensity was accomplished using VAS. Patients were asked to mark maximum pain level experience in the last 2 days. The follow-up assessments were carried out at 4 and 8 weeks after the initiation of treatment. In follow-up assessments of the present study, patients were asked to state the percentage of adherence to the home program and to indicate any adverse effects (this question was asked of the experimental group only) every week.

### 2.4. Statistical Analysis

#### 2.4.1. Sample Estimation

Power estimates were based on a standard deviation of 9, which was obtained from a recent published research work with the same design [31]. It was indicated that assuming a drop-out rate of 30% with a sample size of 20 participants per group would allow differences 10 points in the DASH scores between the experimental and control groups to be detected with >80% power [38].

#### 2.4.2. Data Analysis

Statistical analyses were performed using SPSS software (release 25.0, Armonk, NY: IBM Corp). Each participant allocated a numerical code to use on questionnaires and forms during the study, also used for recording data in SPSS. Continuous data were presented as descriptive statistic means and standard deviations (SD), and categorical data were presented as frequencies. A chi-square test or Fisher's exact test (as appropriate) was used to examine significant differences in categorical variables between the experimental and control groups. At baseline, data were checked for normality and most of the variables were not normally distributed. Therefore, the nonparametric analysis was conducted. The Mann–Whitney Test used to examine significant differences in continuous variables between the experimental and control groups. The Wilcoxon signed rank test was used to examine significant differences in continuous variables between baseline, week 4, and week 8 in both groups. Repeated Measure Analysis of variance (ANOVA) was carried out to compare the dependent variables of pain intensity; shoulder function; internal and external rotation strength; and AROM of forward flexion, abduction, and internal and external rotation between the experimental and control groups at baseline, after 4 weeks, and after 8 weeks of intervention. The null hypothesis (Ho) reported that the addition of handgrip-strengthening exercises to a standardized conventional treatment for SAIS will produce superior results in terms of function, pain reduction, muscle strength, and AROM of the shoulder compared with conventional treatment alone was rejected, and the alternative hypothesis (Ha) that the addition of handgrip-strengthening exercises has no additional benefit was accepted.

## 3. Results

A total of 58 patients with primary SAIS were randomly allocated in this study. Out of them, only 34 SAIS patients completed conventional treatments and handgrip-strengthening exercise interventions for 8 weeks. Descriptive statistics for the demographics and clinical characteristics of the SAIS patients who completed physical therapy treatment interventions as shown in Table 2.

In this study, an identical similarity in baseline outcomes was identified when patients were treated with conventional interventions (control group) compared to those treated with handgrip-strengthening exercise interventions (experimental group). However, a significant difference in the VAS, DASH score, the strength of external rotator, and pain-free AROM of forwarding flexion (*p* < 0.05) were reported in handgrip-treated SAIS compared to those treated with conventional treatments (Table 2).

### 3.1. Adherence to a Physical Therapy Session and Home Program Exercises during Clinical Interventions

**Figure 5:**
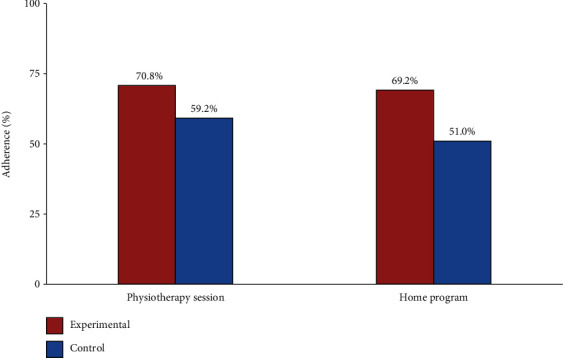
Difference in adherence to physical therapy sessions, home program, and adverse effect of handgrip-strengthening exercises. The data expressed as %. *p* value is significant at the <0.05 level (2-tailed).

To evaluate the adverse effects of handgrip-strengthening exercises, the adherence to supervised (physical therapy session) and unsupervised (home program) exercises training for both clinical interventions was reported in both control and experimental groups (Figure 5). The results showed no significant difference between conventionally treated and handgrip-treated SAIS patient groups (*p* > 0.05), whereas adherence to physiotherapy sessions was*p* = 0.193and adherence home program was*p* = 0.067. Only one patient in the experimental group mentioned that she has pain and heaviness during and after handgrip-strengthening exercises.

### 3.2. Effectiveness of Conventional and Handgrip-Strengthening Treatment Programs

**Figure 6:**
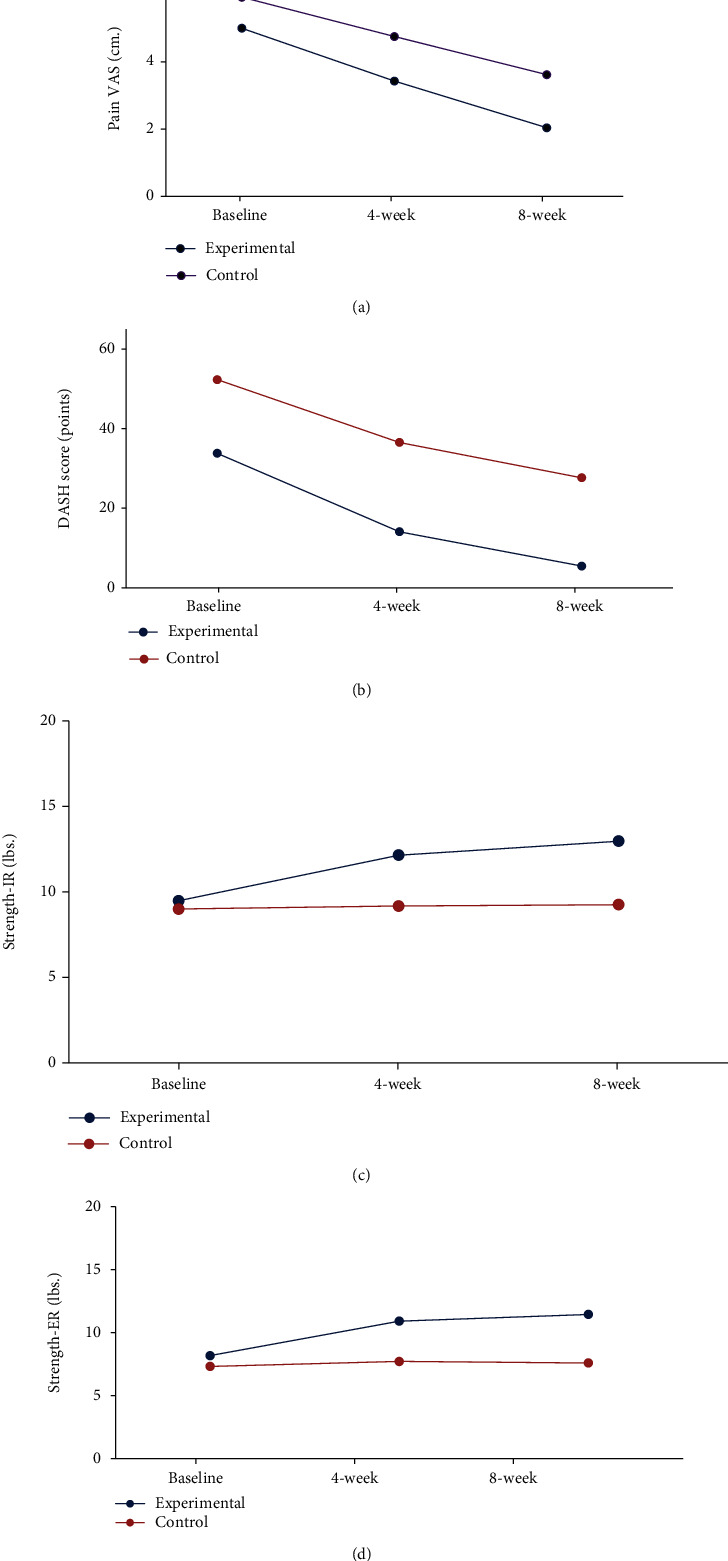
Improvement in shoulder function, muscle strengths, pain, and pain-free active range of motion (AROM) in patients with primary SAIS treated with handgrip-strengthening exercise (experimental group) compared to patients treated with conventional physical therapy interventions (control patients group). Repeated measure ANOVA adjusted for age and gender showed significant improvements in pain (a), shoulder function (DASH score) (b), muscle strength IR (c), and ER (d) for with primary SAIS patients treated with handgrip-strengthening exercise at respective time intervals (4 weeks and 8 weeks), respectively, compared to patients treated with conventional physical therapy interventions (controls). The improvements in the syndromes of primary SAIS are time trend.

**Figure 7:**
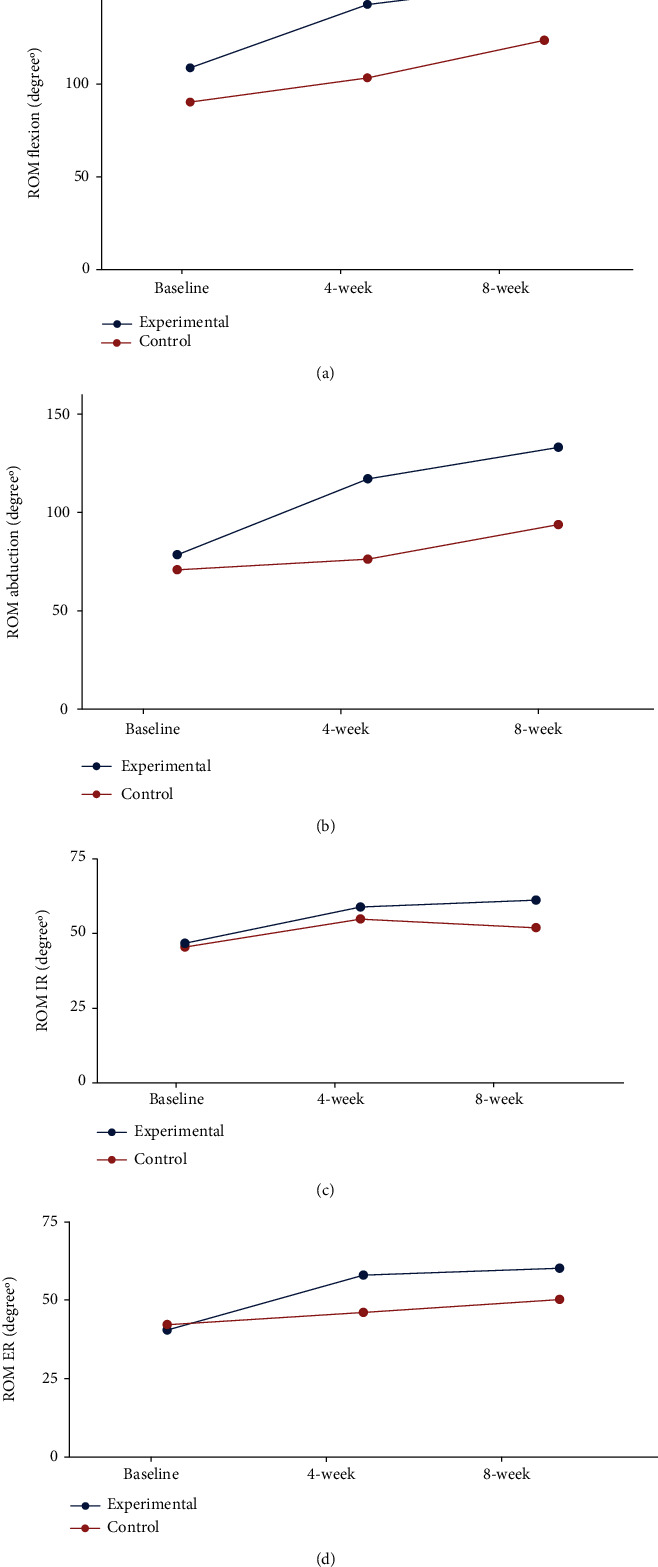
Changes in pain-free active range of motion (AROM) in patients with primary SAIS treated with handgrip-strengthening exercise (experimental group) compared to patients treated with conventional physical therapy interventions (control patients group). Active range of motions (AROM) of forward flexion and abduction of the rotator cuff (RC) muscles were significantly improved following handgrip-strengthening exercise interventions for 8 weeks compared to those treated with conventional physical therapy interventions (a, b). However, SAIS patients of both treating interventions (experiment and control groups) showed no significant difference in active range of motions (AROM) for both internal and external rotations of the rotator cuff (RC) muscles at respective treating time intervals (4 weeks and 8 weeks) (c, d).

In this study, primary SAIS patients treated with handgrip-strengthening exercise showed significant improvement in shoulder function, muscle strengths, pain, and pain-free active range of motion (AROM). Compared to control SAIS patients, primary SAIS patients treated with handgrip-strengthening exercise for 8 weeks showed significant improvements in shoulder function, muscle strengths, pain, and pain-free active range of motion (AROM) except pain-free ROM of both external rotation (ER) and internal rotation (IR) of the rotator cuff (RC) muscles as shown in Table 3 and Figures 6 and 7.

In Figure 6, an improvement in shoulder function, muscle strengths, pain, and pain-free active range of motion (AROM) was reported in patients with primary SAIS treated with handgrip-strengthening exercise (experimental group) compared to patients treated with conventional physical therapy interventions (control patients group). Repeated measure ANOVA adjusted for age and gender showed significant improvements in pain [3A], shoulder function (DASH score) [3B], muscle strength IR [3C], and ER [3D] for with primary SAIS patients treated with handgrip-strengthening exercise at respective time intervals (4 weeks and 8 weeks), respectively, compared to patients treated with conventional physical therapy interventions (controls). The improvements in the syndromes of primary SAIS are time trend.

In this study, changes in pain-free active range of motion (AROM) were reported also in patients with primary SAIS treated with handgrip-strengthening exercise (experimental group) compared to patients treated with conventional physical therapy interventions (control patient group). Active range of motions (AROM) of forward flexion and abduction of the rotator cuff (RC) muscles were significantly improved following handgrip-strengthening exercise interventions for 8 weeks compared to those treated with conventional physical therapy interventions (Figure 4(b) and 7(a)). However, SAIS patients of both treating interventions (experiment and control groups) showed no significant difference in active range of motions (AROM) for both internal and external rotation of the rotator cuff (RC) muscles at respective treating time intervals (4 weeks and 8 weeks) (Figures 4(c) and 7(d)).

### 3.3. Effectiveness of the Treatment Duration

**Figure 8:**
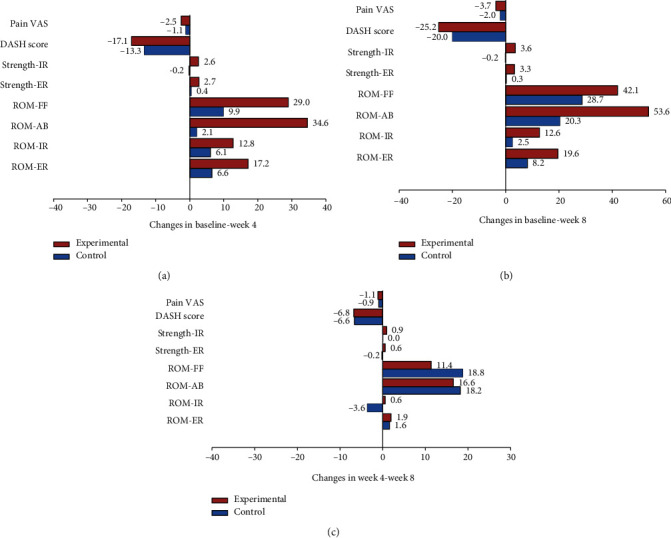
Effectiveness of the treatment duration on the efficiency of both conventional and handgrip-strengthening exercise interventions to improve the primary SAIS syndromes. The results showed all come out clinical measures, VAS, DASH, muscle strength, and pain-free active ROM measures were significantly improved over scheduled time of treatment. The clinically improved measures are significantly correlated at time intervals; 4 weeks (a), 8 weeks (b), and 4–8 weeks (c) compared to baseline data which concluded that treatment of primary SAIS syndromes with conventional interventions either alone or with handgrip-strengthening exercise interventions during 8 weeks is time-dependent.

The improvements of clinical outcome measures: VAS, DASH, muscle strength, and pain-free active ROM, of patients with primary SAIS in conventionally treated patients (control group) and handgrip-strengthening exercise-treated patients (experimental group) were shown to be time-dependent as shown in Table 4 and Figure 8. The data showed that shoulder function, muscle strengths, pain, and pain-free active range of motion (AROM) were significantly improved in both treated primary SAIS patient's groups except for the strength of external rotation, the strength of internal rotation, ROM of external, and ROM of internal rotation as in Table 4. The comparisons revealed that all the differences in all primary SAIS outcome scores are significantly correlated and that treatment with both conventional and handgrip-strengthening exercise treatments is time-dependent (Figure 8).

The results showed that all clinical measures: VAS, DASH, muscle strength, and pain-free active ROM, were significantly improved over scheduled time of treatment. The clinically improved measures are significantly correlated at time intervals; 4 weeks (Figure 8(a)), 8 weeks (Figure 8(b)), and 4–8 weeks (Figure 8(c)) compared to baseline data which concluded that treatment of primary SAIS syndromes with conventional interventions either alone or with handgrip-strengthening exercise interventions during 8 weeks is time-dependent.

## 4. Discussion

In this study, a total of 38 patients with primary SAIS who matched with the proposed inclusions participated in this single-blind randomized clinical trial (SBRCT). Patients were classified into two groups: patients treated with conventional therapy (conventional group) and patients treated with conventional treatments and handgrip-strengthening exercise interventions. All patients completed the proposed treatments for 8 weeks. In addition, patients of the two groups were asked to perform the same respective stretching and HGSE exercises (added to experimental group) at home once a day for eight weeks.

The data showed that treatment with handgrip-strengthening exercises along with conventionally therapy interventions significantly improved primary SAIS in terms of improvement of shoulder function, pain, strength, and pain-free active range of motion (AROM) in patients with primary SAIS. Both treatment programs were effective in decreasing pain, functional disability, and pain-free active ROM of forwarding flexion of the shoulder joint in patients with primary SAIS. More enhancement in shoulder function, pain, strength, and pain-free active range of motion (AROM) was reported in primary SAIS patients treated with handgrip-strengthening exercises, respectively, for 8 weeks. In addition, the results showed that the improvement was time-dependent, whereas four weeks of handgrip-strengthening exercises were sufficient for improving all outcome measures, in which the program was focusing on further improvement in rotator cuff strength by handgrip-strengthening exercises.

Previous research studies suggested that in order to differentiate between subjects with SAIS who had significantly improved shoulder pain versus those who remained stable, the minimally clinically important change (MCIC), for VAS, should be at least 1.4 cm [39]. On the other hand, the MCIC for DASH score should be at least 10 points [40].

In the current study, the mean improvement of VAS score in the experimental group was statistically significant and clinically relevant (mean improvement = −3.69 > MCIC for VAS score) while it was in the control group (mean improvement = −2.01). Moreover, the mean improvement of the DASH score in the primary SAIS who treated with handgrip-strengthening exercise was also clinically relevant (mean improvement = −25.16 > MCIC for DASH score) while it recorded a mean improvement (−19.96 > MCIC for DASH score) among conventionally treated primary SAIS patients.

The results of this study suggest that the addition of handgrip-strengthening exercises to conventional treatment is more effective than the conventional treatment alone in terms of decreasing pain and shoulder disability and improving pain-free active ROM and muscle strength, which support the study's hypothesis. Several components in handgrip-strengthening exercises may contribute to these positive results in a way which handgrip strength influence the rotator cuff. Firstly, the neurological connection was between handgrip and RC. Simply, during handgrip-strengthening exercises, the brain would facilitate the RC muscles to turn on for the arm to function properly and avoid injury. Therefore, the strong handgrip would be to increase the neural drive from hand and shoulder. In previous clinical works, it was found that concurrent activation of indirect propriospinal pathways in handgrip-strengthening exercises provides adaptable movement control allowing integration of sensory information from the shoulder and hand [28, 40, 41].

In addition, handgrip-strengthening exercises were shown to help in the activation of rotator cuff (RC) muscles and decrease activation of the middle and anterior portion of the deltoid. Therefore, when an exercise-based therapy was applied to treat patients with SAIS, targeting rotator cuff muscles with minimal involvement of the deltoid muscles will be effective [42–44]. Similarly, the effects of arm posture and handgrip on shoulder muscle activity during both isometric and dynamic conditions were evaluated previously in healthy subjects [44–46]. In these studies, the electromyography was collected from shoulder muscles (anterior, middle, and posterior fibers of deltoid and infraspinatus) using three-way interactions of shoulder plane, shoulder angle, and handgripping. Their findings suggested that gripping led to a decrease of anterior and middle deltoid activity and an increase in posterior deltoid and infraspinatus activity in all shoulder planes. This confirms our results that handgrip-strengthening exercises may be helpful for patients with SAIS, where the deltoid activation increased and became dominant; therefore, the RC cannot perform the stabilizing action well [44–46].

The progression and positioning of handgrip-strengthening exercises were designed in this study based on previous EMG studies that found that the handgripping was accompanied by a change in excitability of rotator cuff in different shoulder position [32, 33]. In these studies, the potential effect of handgripping on the activity of supraspinatus muscle in SAIS patients was reported and the findings have shown that the amplitude of supraspinatus activity was greater with adding 50% of maximum voluntary contraction of handgrip in a different angle of shoulder abduction (30°, 60°, and 90°) in patients with shoulder impingement syndrome [33].

In the current study, the maximum improvement was in the first 4 weeks when the patient performed the handgrip strengthening exercises in 30° and 60° shoulder abduction position where the maximum decreasing of RC activation may have happened. Our finding is consistent with others [47], who compared the potential muscle activity of the middle deltoid and rotator cuff muscles during abduction in the scapular plane in subjects with shoulder impingement and healthy subjects. In the impingement group, the infraspinatus and subscapularis demonstrate decreased activity during the 30 to 60-degree arc [47]. Later, both a rotator cuff muscle coactivation and middle deltoid muscle activation were significantly measured during elevation of the arm in subjects with shoulder impingement and control subjects [48]. Matching with our results, the patients with impingement in these studies [47, 48] showed significantly decreased supraspinatus, infraspinatus, and subscapularis coactivation and increased middle deltoid activation from 0° to 30° degrees of elevation, while supraspinatus and infraspinatus coactivation decreased from 30 to 60 degrees of elevation; and there is higher coactivation of all rotator cuff muscles from 90 to 120 degrees of elevation of the arm as compared to the healthy subject [48].

Previous studies showed an association between the clinical manifestation of primary SAIS and a decreasing in both rotator cuff strength and shoulder function, which result in the changes in deltoid activation and altered GHJ stability [17, 49, 50]. So, the exercise to strengthen RC is often desired with decreasing the chance of impinging of the RC tendon [22, 34, 51]. Because of the pain, there is typically 90°-120° of humeral elevation; the patient may not be able to do the direct strengthening exercises to RC muscles within this range to reach the maximum RC muscle activation [48]. Besides, it was found that the abduction and external rotation exercises were the most effective to strengthen RC muscles but those may increase the symptoms due to increasing deltoid activation during the performance [52, 53].

The finding of our study showed that the handgrip-strengthening exercises not only improved RC strength (internal and external rotator muscles) but also improved most of outcome measures in greater extent compared to conventionally treated SAIS patients. Indefinitely, our results were supported by the studies, which say that strengthening exercises for rotator cuff muscles can optimize the therapeutic effect on the patient with primary SAIS and minimize associated symptoms [20, 21, 54, 55]. Our results suggest that the influence of the handgrip strengthening exercises on primary SAIS may due to its effect on RC strength. This result is consistent with previous studies that had shown a positive relation between RC strength and handgrip strength [25, 26, 28, 54–56]. In these confirming studies, the effects of handgrip-strengthening exercises on shoulder internal rotation and external rotation peak torque were reported for healthy subjects.

The program used for investigation consisted of handgrip-strengthening exercise by GD Grip Pro (has strength adjusting function) 10 times for three sets twice a day. After 4 weeks, they found that a participant showed significant improvement in external rotation peak torque which significantly matched with our results at respective time intervals: 4 weeks and 8 weeks, respectively. Thus, the adding of handgrip-strengthening exercises in treating interventions could be effective for pain reduction and improvement in functional disability, strengthen rotator cuff muscles, and activate ROM of the shoulder for the patient with primary SAIS.

In addition, our study reported that the treatment with handgrip-strengthening exercises along with conventional interventions is time-dependent. Since the maximum result of pain reduction and improvement in function, RC strength, and pain-free active ROM were attained in week 4 only in experimental group, the authors recommend the addition of handgrip-strengthening exercises to conventional treatment if reduction of pain and improving RC strength, shoulder function, and active ROM of shoulder were the goals of treatment. Thus, in this cases, the treatment frequency and duration remain a source of debate in the literature. In the present study, the treatment sessions of primary SAIS took place twice a week for 8 weeks, which is consistent with previous recommendations [32, 57–59].

In this study, the effects of hand dominance and gender specificity were evaluated in primary SAIS patients conventionally treated with handgrip-strengthening exercises. The results obtained showed that only one-quarter of participants were of their dominant side when treated with handgrip-strengthening exercises. The handgrip-strengthening exercises for this group were a challenge in order to weaken muscle strength and controlling of movement. On the other hand, half of the participants with conventionally treated interventions have a primary SAIS on their dominant side. Therefore, the exercises for a nondominant hand do more than training for the hand; it is also training for the brain. Our data are line with those who reported that the handgrip strength is significantly stronger in a dominant side and controls force better, but there are no differences between sides left or right [9, 60].

Gender has important roles in the quality of life and extent of shoulder pathology. Female candidates for rotator cuff-related surgeries reported more emotional difficulties [61]. In the current study, most of participants were female that may affect our finding. Usually, the risk of sustaining certain pathology and vulnerability health life increased in female more than in male. It was reported in previous studies that the biological differences in anatomy, hormones, aerobic capacity, and strength may significantly affect their clinical improvement [62, 63]. Also, the women with rotator cuff pathology suffer from higher levels of disability and gender qualities contribute to these differences [64].

Finally, our results recommend that treatment with handgrip-strengthening exercises for 4 weeks could be sufficient for pain reduction strength rotator cuff muscles and improvement in functional disability and active ROM of the shoulder. Such finding may help clinicians by getting greater improvement during shorter recovery time. Finally, treatment with handgrip-strengthening exercises for four weeks significantly reduced the pain of the strength rotator cuff muscles and improved both functional disability and active ROM of the shoulder. These findings might be helpful for both patients and health care providers by getting greater improvement of the SAIS patients within a shorter recovery time with minimum expenses whereas the adjustable handgripper is a low-priced exercise therapy.

To the best of the authors' knowledge, this RCT is the first to evaluate the relative effect of handgrip-strengthening exercises in the treatment of primary SAIS. Both treatment programs were effective. However, patients treated with handgrip-strengthening exercises for 8 weeks showed significantly less shoulder pain with more improvement of shoulder function than those who received only conventional treatments.

Finally, improvement in rotator cuff muscles strength was reported more in patients who were treated with handgrip-strengthening exercises. This might give more concentration on handgrip stretching exercise therapy in the future nondrug trials against shoulder pain.

There are some limitations to consider in the present study. It was not possible to blind the patient and the therapist, and so only the assessor was blinded in the current study. Although the adherence to supervised (physical therapy session) and unsupervised (home program) exercises training in both conventional therapy and handgrip-treated SAIS patient groups showed no significant difference, the study utilized self-reported measures, which might be prone to recall bias with larger samples.

## 5. Conclusion

Adding handgrip-strengthening exercises to conventional intervention increases the efficacy of treatment for patients with primary SAIS in terms of shoulder function, pain, muscle strength, and AROM. Thus, future research should also explore the components and parameters that should be included in handgrip strengthening exercise program in order to achieve maximal effects.

## Figures and Tables

**Table 1:** Demographics and clinical characteristics: VAS, DASH, muscle strength, and pain-free active ROM measures, of patients with primary SAIS.

Characteristics	Control (*N* = 16)	Experimental (*N* = 18)	Total (*N* = 34)	*p* value^∗∗^
Age (years)	39.15 ± 7.60	39.05 ± 8.47	39.10 ± 7.94	0.969
Gender (M/F)	4/12	4/14	8/26	0.99
SAIS affected side				
Right	8 (40.0%)	7 (35.0%)	15 (37.5%)	0.744
Left	12 (60.0%)	13 (65.0%)	25 (62.5%)	
Hand dominancy				
No	9 (45.0%)	14 (70.0%)	23 (57.5%)	0.110
Yes	11 (55.0%)	6 (30.0%)	17 (42.5%)	
Working status				
No	10 (50.0%)	8 (40.0%)	18 (45.0%)	0.525
Yes	10 (50.0%)	12 (60.0%)	22 (55.0%)	
Pain VAS	6.21 ± 1.09	5.18 ± 1.06	6.2 ± 1.52	0.006
Shoulder function DASH score	54.22 ± 12.89	35.85 ± 16.97	45.04 ± 14.93	0.001
Strength-Internal rotation	9.54 ± 3.52	9.83 ± 2.31	9.7 ± 2.9	0.370
Strength-External rotation	7.14 ± 1.70	8.56 ± 2.51	7.85 ± 2.3	0.017
Range of motion-Forward flexion	93.45 ± 38.19	116.90 ± 30.98	105.17 ± 34.6	0.027
Range of motion-Abduction	72.70 ± 34.58	90.08 ± 35.17	81.39 ± 34.87	0.072
Range of motion-Internal rotation	47.68 ± 21.19	52.05 ± 18.81	49.86 ± 19.6	0.351
Range of motion-External rotation	48.10 ± 22.15	50.28 ± 24.89	49.19 ± 23.52	0.735

Data obtained expressed as the mean ± standard deviation or number and percentage. Chi-square test or Fisher's exact test was used. *p* value is significant at the <0.05 level (2-tailed). VAS: Visual Analog Scale; DASH: Disability of Arm, Shoulder, and Hand questionnaire.

**Table 2:** The steps of stretching exercises performed in conventional therapy.

Stretching exercises	Descriptions	Figures
Posterior shoulder stretch (10 × 10 sec hold)	Patients pull the elbow across the body with the opposite arm until a stretch is felt at the back of the shoulder.	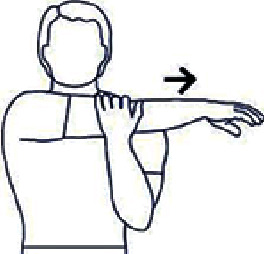
Pectoralis stretch (10 × 10 sec hold)	Patients place their forearm against a wall or door frame and turn the body and feet away from the arm until a stretch is felt in the chest. The elbow or height of arm on the wall may be lowered if pain is experienced.	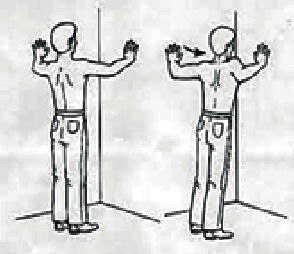
Seated thoracic spine extension (10 × 10 sec hold)	Patients sit in a chair with back support, place a rolled towel at the level of the bottom of the shoulder blades, cross arms over the chest, and arch the back over the towel roll as far as possible without pain.	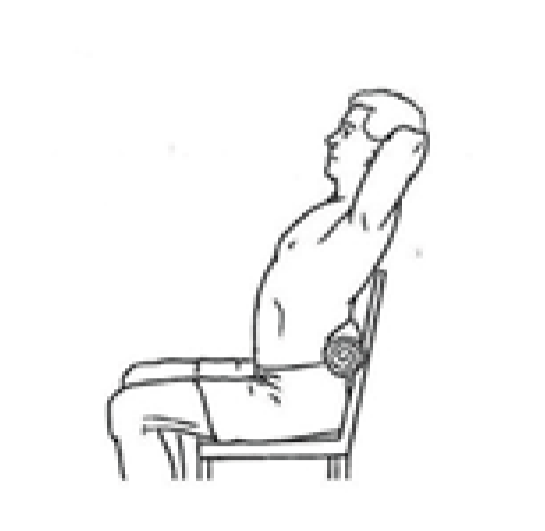
Sleeper stretch (10 × 10 sec)	Patients sleep in a side-lying position with the arm in front of the shoulder and elbow flexed at 90° and use the other arm to push the forearm toward the floor until a stretch is felt in the back of shoulder.	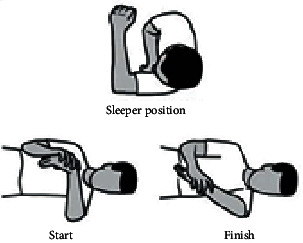

**Table 3:** Estimated adjusted^∗^ marginal means (and standard error) of outcome measures using repeated measure ANOVA.

	Control	Experimental	Mean difference^∗∗^	*p* value^1^ (2-tail)	*p* value^2^ (2-tail)
Pain VAS	4.62 ± 1.12	3.07 ± 1.15	−1.55 ± 1.19	0.015	0.119
Shoulder function-DASH score	40.65 ± 1.19	13.25 ± 1.19	−27.40 ± 1.26	0.001	0.022
SIR	9.39 ± 1.04	11.83 ± 1.04	2.44 ± 1.06	0.001	0.027
SER	7.64 ± 1.05	10.35 ± 1.05	2.71 ± 1.07	0.001	0.013
ROM-FF	101.80 ± 1.07	131.11 ± 1.06	29.31 ± 1.09	0.002	0.007
ROM-A	76.63 ± 1.09	104.17 ± 1.08	27.54 ± 1.11	0.004	0.005
ROM-IR	48.18 ± 1.07	53.36 ± 1.07	5.17 ± 1.09	0.126	0.318
ROM-ER	40.98 ± 1.11	47.99 ± 1.10	7.01 ± 1.15	0.128	0.050

Data expressed as the mean ± SD. ^∗^Adjusted for age and gender. ^∗∗^Difference was defined as experimental minus control group. Wilcoxon signed rank test and Repeated Measure Analysis of variance (ANOVA). *p* value^1^ indicates significant differences between groups. *p* value ^2^ indicates significant differences in time trend. VAS: Visual Analog Scale; DASH score: Disability of Arm, Shoulder, and Hand questionnaire; SIR: Strength-Internal rotation; SER: Strength-External rotation; ROM-FF: Range of Motion-Forward flexion; ROM-A: Range of Motion-Abduction; ROM-IR: Range of Motion-Internal rotation; ROM-ER: Range of Motion-External rotation.

**Table 4:** Effectiveness of the treatment duration on clinical characteristics: VAS, DASH, muscle strength, and pain-free active ROM measures, of patients with primary SAIS in conventional treated patients (control group) and handgrip-strengthening exercise treated patients (experimental group).

Clinical characteristics	Mean ± SD			*p* value (2-tail)		
	BL	wk 4	wk 8	BL–wk 4	wk 4–wk 8	BL–wk 8
Pain VAS						
Experimental group	5.18 ± 1.06	2.43 ± 2.14	1.29 ± 1.96	<0.001	0.004	<0.001
Control group	6.01 ± 1.12	4.91 ± 1.23	4.00 ± 1.85	0.007	0.030	0.003
Shoulder function-DASH score						
Experimental group	35.85 ± 16.97	17.69 ± 14.27	10.69 ± 16.37	<0.001	0.008	<0.001
Control group	53.85 ± 12.29	40.52 ± 18.09	33.89 ± 19.60	0.009	0.017	0.003
Strength-Internal rotation						
Experimental group	9.83 ± 2.31	12.41 ± 2.54	13.32 ± 3.23	<0.001	0.062	<0.001
Control group	9.48 ± 3.48	9.28 ± 1.43	9.31 ± 1.10	0.319	0.345	0.308
Strength-External rotation						
Experimental group	8.56 ± 2.51	11.29 ± 2.75	11.82 ± 3.22	<0.001	0.139	<0.001
Control group	7.50 ± 1.39	7.91 ± 1.60	7.70 ± 1.34	0.330	0.356	0.335
Range of motion-Forward flexion						
Experimental group	116.90 ± 30.98	145.88 ± 23.61	156.36 ± 22.10	<0.001	0.008	<0.001
Control group	97.69 ± 39.07	107.59 ± 30.06	126.36 ± 26.52	0.128	0.002	0.005
Range of motion-Abduction						
Experimental group	90.08 ± 35.17	124.68 ± 33.74	138.31 ± 33.67	<0.001	0.002	<0.001
Control group	78.00 ± 36.08	80.06 ± 24.39	98.28 ± 29.91	0.213	0.001	0.009
Range of motion-Internal rotation						
Experimental group	52.05 ± 18.81	64.83 ± 20.11	62.44 ± 13.12	0.001	0.197	0.005
Control group	50.31 ± 22.59	56.42 ± 13.46	52.81 ± 9.59	0.219	0.190	0.255
Range of motion-External rotation						
Experimental group	50.28 ± 24.89	67.43 ± 28.28	65.25 ± 24.56	<0.001	0.149	0.001
Control group	46.81 ± 21.40	53.38 ± 27.16	54.97 ± 22.67	0.052	0.123	0.032

Data expressed as the mean ± SD. Wilcoxon signed rank test and Repeated Measure Analysis of variance (ANOVA).

## Data Availability

All data generated or analyzed during this study are presented in the manuscript. Please contact the corresponding author for access to data presented in this study.
